# Dysentery

**Published:** 1888-07

**Authors:** 


### EDITORIALS AND MISCELLANEOUS.

DYSENTERY.—The season for dysenteries and summer diarrhœas is upon us. In the early spring and summer the economy does not at once adapt itself to the changed conditions of the weather. The heat of day contrasts strongly with the night, and the warmth of the sun stimulates the skin, increasing the perspiration, which by the cool nights and damp mornings of spring is liable to sudden checks, interfering with the equilibrium existing between the enunctories, tending to congestions of the liver and hyperæmia of the intestinal mucous membrane, while the indulgence in the use of berries, garden peas, and other vegetables, in this predisposed condition, irritate the bowels.

A little caution and circumspection might prevent the trouble, but few think of this, and so the doctor obtains his summer crop of this kind of practice.

Ordinarily a diarrhœa relieves itself, and need not be interfered with. If it prove excessive, an opiate may be sufficient to arrest it; but dysentery had best be looked after. In a vast majority of cases it is well to convert a dysentery into a diarrhœa at the outset by the use of salines, with which an opiate may be combined, if there is much distress or griping.

℞. Epsom salts........................................1 tablespoonful,

Water........................................½ pint,

Paregoric........................................2 teaspoonsful.

M. Give a tablespoonful of this mixture every hour until the character of the discharges change, then give at longer intervals for twelve or twenty-four hours; then check the bowels with a decided opiate, using at the same time a large cornmeal poultice upon the abdomen, sprinkled with mustard. In many cases this will suffice to relieve the patient.

Another method which we often use successfully, especially in cases of a bilious character with considerable fever, is as follows:

℞. Calomel........................................gr. vi,
Ipecac........................................gr. iij,
Morph........................................gr. ⅓.

M. and make three powders. One every two hours. Put on mustard poultice as in the other plan, and give for the fever the following:

℞. Water........................................℥ iv,

Aconite........................................gtts. vi.

M. Take one teaspoonful every hour until fever cools down. The powders will act after a few hours, causing two to four bilious, discharges when the checking process may be attempted with morphine and ipecac, each ⅓ gr., repeated if necessary in two to four hours.

